# Arterial spin labeling MRI applied to migraine: current insights and future perspectives

**DOI:** 10.1186/s10194-023-01597-y

**Published:** 2023-06-16

**Authors:** Antonio Russo, Marcello Silvestro, Alessandro Tessitore, Ilaria Orologio, Alessandro Pasquale De Rosa, Rosa De Micco, Gioacchino Tedeschi, Fabrizio Esposito, Mario Cirillo

**Affiliations:** 1 Headache Centre, Department of Advanced Medical and Surgical Sciences, University of Campania “Luigi Vanvitelli”, Naples, Italy; 2 Advanced MRI Neuroimaging Centre, Department of Advanced Medical and Surgical Sciences, University of Campania “Luigi Vanvitelli”, Naples, Italy

**Keywords:** Migraine without aura, Migraine with aura, Arterial spin labelling, MRI, Cerebral blood flow

## Abstract

**Introduction:**

Advanced neuroimaging techniques have extensively contributed to elucidate the complex mechanisms underpinning the pathophysiology of migraine, a neurovascular disorder characterized by episodes of headache associated with a constellation of non-pain symptoms. The present manuscript, summarizing the most recent progresses of the arterial spin labelling (ASL) MRI techniques and the most significant findings from ASL studies conducted in migraine, is aimed to clarify how ASL investigations are contributing to the evolving insight on migraine pathophysiology and their putative role in migraine clinical setting. ASL techniques, allowing to quantitatively demonstrate changes in cerebral blood flow (CBF) both during the attacks and in the course of interictal period, could represent the melting point between advanced neuroimaging investigations, conducted with pure scientific purposes, and conventional neuroimaging approaches, employed in the diagnostic decision-making processes.

**Main body:**

Converging ASL evidences have demonstrated that abnormal CBF, exceeding the boundaries of a single vascular territory, with biphasic trend dominated by an initial hypoperfusion (during the aura phenomenon but also in the first part of the headache phase) followed by hyperperfusion, characterizes migraine with aura attack and can represent a valuable clinical tool in the differential diagnosis from acute ischemic strokes and epileptic seizures.

Studies conducted during migraine without aura attacks are converging to highlight the involvement of dorsolateral pons and hypothalamus in migraine pathophysiology, albeit not able to disentangle their role as “migraine generators” from mere attack epiphenomenon. Furthermore, ASL findings tend to support the presence of perfusion abnormalities in brain regions known to be involved in aura ignition and propagation as well as in areas involved in multisensory processing, in both patients with migraine with aura and migraine without aura.

**Conclusion:**

Although ASL studies have dramatically clarified quality and timing of perfusion abnormalities during migraine with aura attacks, the same cannot be said for perfusion changes during migraine attacks without aura and interictal periods. Future studies with more rigorous methodological approaches in terms of study protocol, ASL technique and sample selection and size are mandatory to exploit the possibility of better understanding migraine pathophysiology and identifying neuroimaging biomarkers of each migraine phase in different migraine phenotypes.

## Introduction

Migraine is a genetically determined neurovascular disorder featuring recurrent episodes of headache accompanied by a constellation of non-pain symptoms including sensory hypersensitivity, neurovegetative symptoms as well as cognitive and emotional changes [1]. A non-negligible percentage of patients (about one third of patients with migraine) experiences focal neurological symptoms (the so-called aura), usually starting before the headache onset, gradually spreading up to slowly disappear. Interestingly, besides the ictal burden, compelling evidences support clinical and psychosocial impact also during interictal periods in these patients [2].

The diagnosis of migraine is based on the different features of headache (e.g., quality, location, duration, timing, severity), the presence of accompanying symptoms as phono/photophobia, nausea and vomiting, along with attacks-related disability and precipitating events [3]. Therefore, due to the unavailability of specific neuroimaging biomarkers, in the current clinical practice, migraine diagnosis is an eminently clinical process where instrumental examinations take a part only in excluding causes of secondary headaches or, eventually, migraine-worsening disorders [4].

In the last decades, among the advanced functional, structural and microstructural neuroimaging approaches, arterial spin labelling (ASL) techniques, able to quantitatively investigate, with clinical high field MRI, both global and local changes in cerebral blood flow (CBF), represented a substantial breakthrough in the knowledge of human brain processes as well as in other brain disorders [5].

Specifically, ASL techniques have contributed to elucidate the complex mechanisms underlying migraine pathophysiology by the identification of CBF abnormalities both during attacks and in the course of interictal period.

Furthermore, while until few years ago the idea that advanced neuroimaging techniques could play a role in the evaluation of patients with migraine in clinical setting was “heretical”, nowadays ASL techniques, by providing quantitative MRI biomarkers, could represent the melting point between advanced neuroimaging investigations, often conducted on a group-level with pure scientific purposes, and conventional neuroimaging approaches, already employed in the diagnostic decision-making process applied to single patients with migraine [6]. Similarly, in a futuristic way, ASL techniques could be putatively able to provide prognostic critical information in order to support “tailored” therapeutic strategies in every single patient with migraine, overcoming the limitations related to the variability of the judgment and experience of clinicians.

The present narrative review focuses on the progresses of the ASL techniques over time, their role in the evolution of understanding migraine pathophysiology and their applicability in migraine clinical setting.

## Methods

Two of the authors (AR and MS) performed searches on the PubMed.com website to identify all the original articles by entering the key word "migraine without aura" or “migraine with aura” combined with "arterial spin labeling". The literature search was finalized on Pubmed.com March 20^th^, 2023 and was restricted to human studies written in English language. Our search approach resulted, after removing duplicates, in a total of 57 papers. Among these, 25 reviews, letters, experiments testing treatment effects or studies not focused on migraine as well as articles in which methods were not properly described were excluded.

To identify additional relevant studies, we thoroughly checked the reference list for each considered article. Finally, the author neurologists (AR, AT and GT) reviewed the papers found in the preliminary phase to screen for inclusion and exclusion criteria. We ended up with a total of 32 peer-reviewed studies.

### Arterial spin labelling

ASL is a non-contrast MRI technique able to perform a measurement of brain perfusion by means of magnetically labelled arterial blood water working as an endogenous freely diffusible tracer [7–9]. Radiofrequency inversion pulses are used to modify the T1 signal longitudinal magnetizing (“label”) moving protons within the carotid and vertebral arteries at the skull base. Image acquisition occurs after a time delay to allow the labeled blood to flow into the cerebral arteries (i.e. arterial capillaries) where imaging slices are acquired. Perfusion characteristics are quantitatively determined by pairwise comparison of imaging data with and without labeling (the latter working as control) [7–9].

The advantages of ASL compared with conventional (e.g. 133Xe-SPECT, H215O-PET) as well as advanced (e.g. phase-contrast MRI) perfusion-based techniques include absolute quantification, repeatability, avoidance of intravenous contrast administration and, overall, superior spatial resolution and sensitivity. More specifically, all the major methods that have been developed for measuring CBF are based on the principles of compartmental modeling and tracer kinetics describing the dynamics of a tracer as it crosses the arterial tree into the brain’s microvasculature (non-diffusible tracers) and into the tissue (diffusible tracers) prior to venous washout. Contrariwise, ASL fMRI uses arterial water as an endogenous tracer and thus does not require injection of exogenous tracers that can be uncomfortable and potentially harmful. Therefore, ASL, being noninvasive, is safe to repeat over time and can be used to track changes in CBF in the course of disease progression or treatment. Furthermore, ASL yields an absolute measurement of CBF and therefore any change in flow can be expressed in physiologically meaningful units rather than as a percentage of change. Finally, ASL yields CBF images with higher spatial and temporal resolution than any other current technique.

Based on the labeling scheme, ASL can be divided into 3 categories: pulsed ASL (pASL), continuous ASL (cASL) and the more recently introduced pseudo-continuous ASL (pcASL).

The pASL was the first approach developed and is characterized by the application of a single inversion pulse providing high and stable labeling efficiency (e.g., up to 95%), although this approach is burdened by a suboptimal SNR and a limited spatial coverage.

Contrariwise, cASL, characterized by the constant application of a pulse gradient to label arterial blood flowing through a given plane, provides a higher SNR (about 30–50% higher than pASL) although with suboptimal labeling efficiency (e.g., up to 68%).

Finally, the pcASL, using a long series of radiofrequency and gradient pulses, provides a more robust measure of CBF, even at low flows, and is able to offer both higher SNR and labeling efficiency compared with pASL and cASL respectively.

In order to maximize the SNR and temporal stability of ASL scans, background suppression techniques and three-dimensional (3D) fast imaging sequences have been developed. 3D acquisitions not only offer increased SNR compared with two-dimensional (2D) acquisitions but they are also ideally suited for background suppression. A recent consensus recommended pcASL with background-suppressed 3D acquisitions as the preferred strategy for ASL clinical applications [5].

It is noteworthy that using the ASL techniques the selection of post-labeling delay (PLD) (that is the time interval between the labeling and the slice acquisition) is crucial to obtain reliable measurements. Specifically, the time interval must be long enough to allow labeled protons to flow into the imaging slice, but not so long that the majority of measurable tracer has decayed. For this reason, until few years ago, the above-mentioned ASL sequences, characterized by a single PLD between the labeling plane and the image acquisition, were burdened by a poor accuracy. More specifically, the variability of regional arterial transit time (ATT), depending on physiological and anatomical parameters (including, e.g., the cardiac output) as well as on the coexistence of some cerebrovascular conditions (e.g., stenotic or low calibre arteries, or collateral circulation), resulted in a potential mismatch between labeling plane (radiofrequency-labeled local blood flow) and 3D image acquisition (PLD sequences) [10]. Another limitation of ASL sequences is related to the fact that settings cannot be easily adjusted or optimized to achieve the highest sensitivity for regional CBF changes, considering single or multiple perfusion territories, within functional relevant domains of interest. This is a momentous issue whenever the highest effects of reduced or increased CBF may encompass adjacent perfusion territories characterized by different ATT or when specific pathological conditions, systematically affecting CBF parameters, may occur.

In order to surmount the above-mentioned constraints a modified ASL technique has been developed, the so-called multi-delay-3D-pcASL, adding to the noteworthy advantages of 3D-pcASL: i) the proficiency of acquiring volumes with multiple different post-labeling delay times within the same series (i.e. multi-delay) increasing the reliability of whole brain CBF analyses ii) the possibility to contextual estimate adjunctive hemodynamic parameters as “mean arterial transit time” (ATT), arterial cerebral blood volumes and visualize the collateral flow through dynamic image series [11]. As a proof of the concept, previous multi-delay-3D-pcASL studies have demonstrated that the use of multiple post-labeling times may lead to sufficiently accurate ATT estimation, thus improving the overall perfusion quantification models and, consequently, providing CBF maps with reduced errors from ATT and tissue T1 variations [12].

Beyond the mere evaluation of regional CBF, ASL techniques allow broader applications to assess brain function during both resting and task condition [13]. Specifically, ASL can be used to explore the brain resting-state functional connectivity (as an alternative way than the blood oxygen level-dependent (BOLD) technique) for a functional connectome analysis), and neurovascular coupling (if CBF and BOLD signals are properly combined from concurrent ASL and BOLD sequences) [14]. The neurovascular coupling is the complex biological process that underlies use-dependent increases in blood flow in response to neural activation.

### ASL application to migraine models

**Fig. 1:**
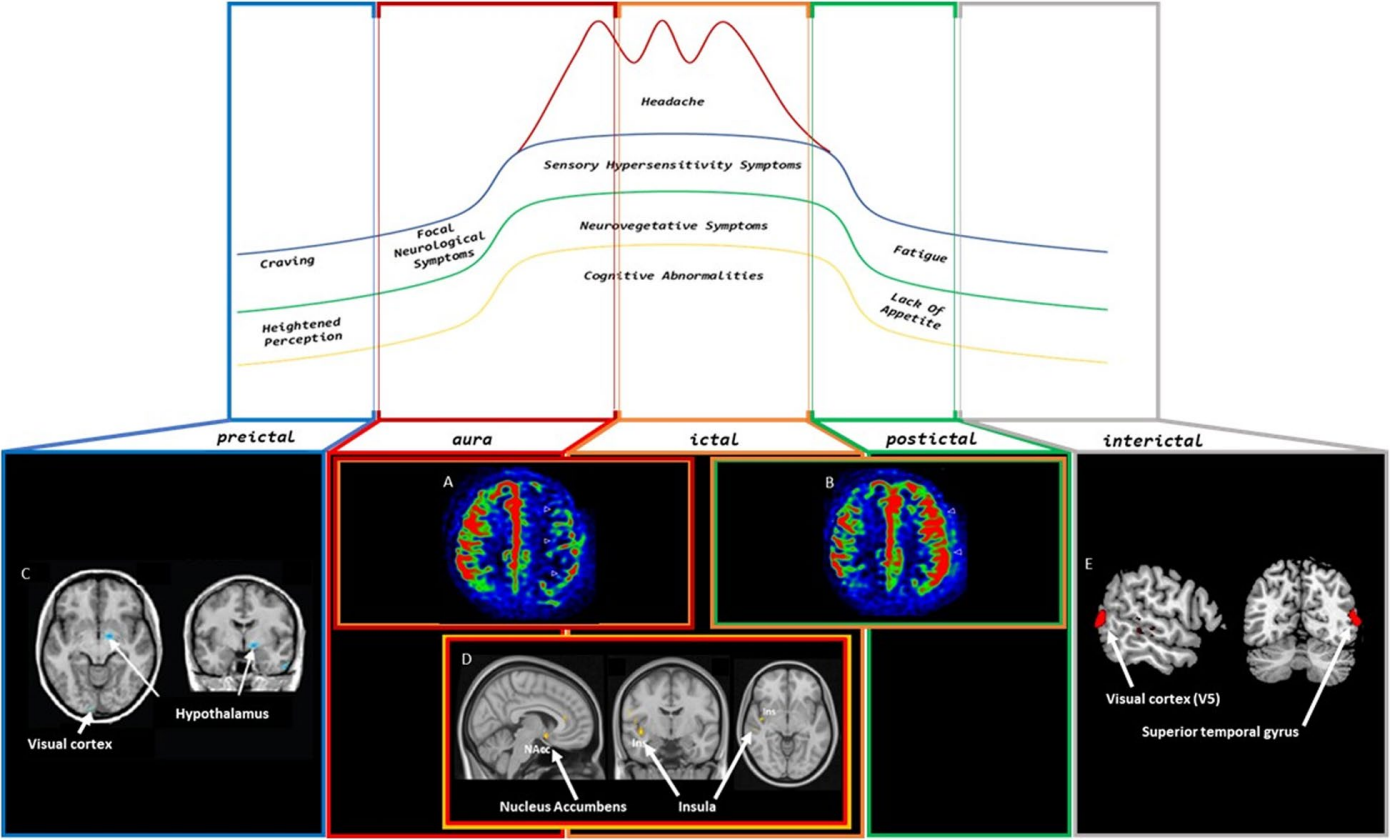
ASL perfusion changes for each phase of migraine cycle. **A** and **B** CBF maps showing an early left hemisphere hypoperfusion followed by hyperperfusion in areas congruous with aura symptoms (exceeding the boundaries of single vascular territories) (modified from Boulouis et al., 2016 [18]). **C** Reduced regional CBF in lateral hypothalamus and visual cortex during the preictal phase of migraine attack (modified from Meylakh et al., 2020 [38]). **D** Increased regional CBF in limbic circuit during the headache phase of migraine (migraine without aura attack) (modified from Stankewitz et al., 2021 [43]). **E** Increased regional CBF in extrastriate cortex and superior temporal gyrus during the interictal period (migraine with aura) (modified from Michels et al., 2019 [28])

It is known that migraine is not simply headache but a complex and multifaceted neurological disease. Although head pain is the most characterizing symptom, patients with migraine also experience a plethora of non-headache symptoms starting before headache onset or persisting after its resolution, classically divided into the preictal, aura, headache and postdromal as well as interictal phases, overall configuring the so-called migraine cycle. In the last years, advanced neuroimaging studies have played a critical role in demonstrating, during the preictal phase but persisting throughout the whole headache timeline, the early activation of the hypothalamus as well as trigeminal, pontine and thalamo-cortical pathways [15]. Along with the elucidation of the involvement of neuronal pathways underpinning each phase of migraine cycle, the ASL techniques have further contributed to clarify the neurovascular substrates of migraine disorder (See Table 1 and Fig. 1 for further information).

**Table 1:** Main features of ASL studies applied to migraine

Reference	N. of patients	Technique	Main findings	Interpretation
Cadiot et al., 2018 [16]	17 MwA pts (median time of 6 h after the symptoms onset)	MRI (including TOF-MRA and 2D pulsed ASL)	- ASL: 16 children presented hypoperfusion in one or more cerebral lobes - TOF-MRA: 12 children presented vasospasm within the intracranial vasculature - 100% of the abnormal TOF-MRA images were associated with homolateral hypoperfusion	The association of perfusion abnormalities on ASL in brain regions, not corresponding to vascular territories, and vascular vasospasm in TOF-MRA, can aid diagnosis of MwA attack
Kim et al., 2016 [17]	1 pt with hemiplegic migraine during attack (R sided)	3 T MRI (including 3D pseudo-continuous ASL and DSC)	- DSC showed hypoperfusion with posterior predominance in the L cerebral hemisphere - ASL perfusion correlated well with DSC findings	In pts with hemiplegic migraine, during an acute migraine attack, hypoperfusion in the affected cerebral hemisphere may be visualized with ASL technique
Boulouis et al., 2016 [18]	10 MwA pts during attack (median onset-to-MRI delay was 12 h)	1.5 T MRI (including 3D pseudo-continuous ASL and DWI)	CBF was: - decreased in a brain region consistent with symptoms when MRI was performed less than 14 h after onset - increased if the MRI was performed 17 h or more after - DWI was normal	ASL provides insight in understanding of pathophysiology of MwA and helps ruling out acute ischaemic stroke
Pollock et al., 2008 [19]	3 migraine pts (1 hemiplegic migraine, 2 with visual aura) between 6 and 24 h from neurological symptoms onset	MRI (including ASL perfusion)	Pt 1: - marked regional hyperperfusion in the R frontal and parietal cortex Pt 2 and 3: - ASL showed hyperperfusion in the medial occipital lobes bilaterally	ASL perfusion analysis may help identify pts with vascular or hemiplegic migraines by showing associated hypoperfusion during the aura phase and hyperperfusion during the headache phase
Burns et al., 2017 [20]	1 pt with CM and MwA during and after aura (R upper limb paresthesia and aphasia)	MRI (including ASL)	- Normal DWI - ASL showed: ◦ hypoperfusion extending over the entire L hemisphere during aura ◦ hyperperfusion over the L parieto-occipital cortex 28 h later (headache still present, aphasia resolved)	ASL allows to detect hypoperfusion during the initial phase of aura extending over multiple vascular territories, and delayed hyperperfusion several hours after the beginning of migraine attack and potentially persisting after resolution of the aura during the headache phase of migraine cycle
Law-Ye et al., 2017 [21]	1 MwA pt (sudden aphasia fellowed by headache) during aura	MRI (including ASL)	- Normal axial T2-FLAIR and DWI - ASL showed an extensive hypoperfusion of the L hemisphere, involving anterior and posterior vascular territories	ASL is especially useful in acute neurological deficits helping to diagnose mimics of stroke such as MwA
Wolf et al., 2017 [22]	4 MwA pts during aura, headache, and asymptomatic phases	MRI (including 3D pseudo-continuous ASL)	- Aura: adjacent hypoperfused and hyperperfused areas not respecting the boundaries of major cerebral vascular territories - During headache: regional hyperperfusion - Asymptomatic phase: normal perfusion	ASL perfusion imaging is a contrast agent-free method suitable for assessment of reversible perfusion changes during or immediately after aura
Cobb-Pitstick et al., 2018 [23]	12 hemiplegic migraine pts between 3 and 11 h from neurologic symptoms onset	MRI (including 3D pseudo-continuous ASL)	All had normal DWI and abnormal ASL findings with differences in cerebral perfusion compared with the unaffected hemisphere: ◦ 9 hypoperfusion ◦ 3 hyperperfusion (indicating the transition to hyperperfusion by 11, 5.5, and 7.5 h)	The alteration of ASL sequences with normal DWI help to distinguishing hemiplegic migraine from stroke
Uetani et al., 2018 [24]	49 migraine pts (33 only migraine; 16 with other neurological symptoms: 8 aura, 3 visual symptoms, 9 motor disabilities, 7 confusion) during attack	3 T MRI (including 3D pulsed ASL)	- 11 pts exhibited perfusion abnormality, especially in the occipital lobe (73%) - no abnormality on non-ASL MRI - Higher prevalence of aura, motor disabilities, confusion, hospitalization in pts with perfusion abnormalities	In paediatric and adolescent pts with migraine, ASL shows a high prevalence of perfusion abnormality, especially in the occipital lobe
Kato et al., 2010 [25]	One MwoA scanned during 2 episodes of migraine: - during attack (within 1 h post-onset) - 30 min after oral administration of rizatriptan 10 mg attack-free period	3 T MRI (including pulsed ASL)	During attack (compared to migraine-free period): - significant relative hypoperfusion in bilateral median thalamic areas and hypothalamus - significant relative hyperperfusion in the frontal cortex 30 min after treatment (compared to images during attack): - relative improvement of perfusion in the bilateral median thalamic areas and hypothalamus	Hypothalamus and its surrounding areas may participate in the pathogenesis of migraine attack
Younis et al., 2021 [26]	26 MwoA pts before and 6 h after administration of CGRP and sildenafil to induce MwoA attacks	3 T MRI (including pseudo-continuous ASL) and proton MR spectroscopy	- CBF increased in dorsolateral pons, ipsilateral to pain side during attacks, compared to outside attacks - Glutamate levels in the same area remained unchanged during attacks, total creatine levels increased	Dorsolateral pontine activation during migraine was not associated with higher glutamate levels, while the concurrently increased total creatine levels may suggest an altered energy metabolism
Gil-Gouveia et al., 2017 [27]	13 MwoA pts during attack and headache-free period	3 T MRI (including 3D pseudo-continuous ASL)	Average total CBF values were similar within and outside the attack	Significant perfusion changes are not expected to occur during MwoA
Michels et al., 2019 [28]	17 migraine pts (13 MwA, 4 MwoA) free from migraine attacks at least 48 h before and after MRI, 19 HC	3 T MRI (including 2D pseudo-continuous ASL)	Hyperperfusion in the extrastriate cortex (area V5) in migraineurs in comparison to HC, more pronounced in MwA during the interictal state	In the interictal state, hyperperfusion was found in the supposed region of CSD onset, located occipito-temporally
Cucchiara et al., 2013 [29]	117 migraine pts (56 MwA, 61 MwoA) 53 HC	3 T MRI (including 3D pseudo-continuous ASL)	- Incomplete circle of Willis was significantly more common in the MwA compared to HC and MwoA - MwA had a higher burden of variants than HC - Pts with an incomplete circle had greater asymmetry in hemispheric CBF - Specific posterior cerebral artery variants were associated with greater asymmetries of blood flow in the posterior cerebral artery territory	An incomplete circle of Willis is more common in MwA pts than HC, and is associated with CBF abnormalities
Puledda et al., 2021 [30]	24 VSS pts (15 with concomitant episodic migraine diagnosis) and 24 HC scanned at rest and during “snow-like” visual stimulus	3 T MRI (including 3D pseudo-continuous ASL)	- Higher rCBF in VSS pts vs HC at rest and during “snow-like” visual stimulus in the cuneus, precuneus, inferior and superior parietal lobule superior parietal lobule, supplementary motor area, frontal eye fields, premotor cortex, posterior cingulate cortex, middle frontal gyrus, angular gyrus, post central gyrus, middle and superior occipital lobule - rCBF increased in R insula in VSS pts vs HC during visual stimulation	Pts with VSS show an increased rCBF in a wide network of intrinsic brain areas involved in the processing of complex sensory and cognitive states independently of the presence of an external visual stimulus, suggesting that these abnormalities could be a causal factor of this disorder
Hodkinson et al., 2015 [31]	17 MwoA pts free from migraine attacks at least 72 h before and 24 h after MRI, 17 HC	3 T MRI (including pseudo-continuous ASL)	- Migraineurs had significantly higher rCBF in both the R and L post-central gyrus compared to HC - CBF values in post-central gyrus were positively correlated with attack frequency, but not the duration of illness or the age of the pts	These results demonstrate the presence of a disease-specific functional deficit in a known region of the trigemino-cortical pathway, which may be driven by adaptive or maladaptive functional plasticity
Youssef et al., 2017 [32]	22 MwoA pts free from migraine attacks at least 72 h before and 24 h after MRI, 22 HC	3 T MRI (including pseudo-continuous ASL)	- Significant resting rCBF increases within bilateral post-central gyrus compared with HC - Within the R post-central gyrus positive correlation between blood flow value with migraine attack frequency and cutaneous allodynia	These alterations within post-central gyrus may be consequence of repeated migraine attacks
Chen et al., 2018 [33]	15 MwoA pts free from migraine attacks at least 72 h before MRI, 15 HC	3 T MRI (including 3D pseudo-continuous ASL)	- Increased CBF in L BA38 - No significantly decreased CBF - HAMD scores significantly positive correlation with the CBF value of the L BA38	Pattern of cerebral hyperperfusion may elucidate the neurogenic mechanism in the MwoA genesis
Fu et al., 2022 [34]	88 migraine pts (32 MwA, 56 MwoA) during interictal period 44 HC	3 T MRI (including 3D pseudo-continuous ASL)	Pts with MwA vs MwoA and HC present: - higher CBF levels in superior frontal gyrus, postcentral gyrus and cerebellum, - lower CBF levels in the middle frontal gyrus, thalamus and medioventral occipital cortex - these variations significantly correlated with clinical rating scales	The alternated CBF in brain regions overlaid important component of neuro-networks and circuits modulating migraine pain processing and showed consistency with previous studies in differentiating MwA from MwoA and HC
Zhang et al., 2021 [35]	40 MwoA pts free from migraine attacks at least 48 h before and after MRI, 42 HC	3 T MRI (including 3D pseudo-continuous ASL)	- MwoA patients had higher CBF in R middle frontal orbital gyrus and R middle frontal gyrus, while that in Vermis declined - Increased CBF positively correlated with VLSQ-8 and monthly attack frequency score - Decreased CBF connectivity between R middle frontal orbital gyrus and L superior frontal gyrus, R putamen, R caudate, R angular gyrus - Increased CBF connectivity between L calcarine cortex and R middle frontal orbital gyrus	MwoA pts exhibit abnormalities in regional CBF and feature CBF connection defects at the RS in areas involve information perception, information integration, and emotional, pain and visual processing
Liu et al., 2022 [36]	13 CM pts (during interictal period) 15 HC	3 T MRI (including 3D pseudo-continuous ASL)	- Lower CBF value in CM pts vs HC in L nucleus accumbens - Negative correlation between CBF value of the L nucleus accumbens and VAS score - No significant difference for the volume of bilateral nucleus accumbens in CM vs HC	Hypoperfusion of the L nucleus accumbens could be considered as a potential diagnostic imaging biomarker of CM pts
Bai et al., 2022 [37]	18 CM during interictal period, 15 NDPH, 15 HC	3 T MRI (including 3D pseudo-continuous ASL)	NDPH vs HC: - decreased CBF and aCBV values in R hemisphere (posterior and middle orbital gyrus, ventral anterior nucleus of thalamus) CM vs HC: - increased CBF and aCBV values in the ventral lateral nucleus of thalamus bilaterally	Being the thalamus a relay centre for ascending nociceptive information, recurring attacks of migraine in CM pts, could lead to increase neuronal thalamic activation and regional CBF
Meylakh et al., 2020 [38]	34 migraine pts (25 MwoA, 9 MwA) - 22 scanned during interictal period (free from migraine at least 72 h before and 24 h after MRI) - 13 scanned immediately following the end of attacks (within 72 h) - 7 scanned prior to an attack (within 24 h) 26 HC	3 T MRI (including pseudo-continuous ASL)	- Prior to an attack, RS-CBF decreased in the lateral hypothalamus - Resting functional connectivity strength decreased between the lateral hypothalamus and regions of the pain processing pathway (midbrain periaqueductal gray, dorsal pons, rostral ventromedial medulla, and cingulate cortex) only before an attack	Altered hypothalamic function and connectivity in the period immediately prior to a migraine headache supports the hypothesis that hypothalamus is involved in migraine attack ignition
Datta et al., 2013 [39]	50 migraine pts (25 MwA, 25 MwoA) during interictal period; 25 HC	3 T MRI (including pulsed ASL and BOLD-fMRI)	BOLD fMRI response to visual stimulation within primary visual cortex and in the lateral geniculate nuclei was: - greater in MwA compared to MwoA and HC No difference between MwoA and HC Increased activation in MWA was confined to the occipital pole - Regional resting cerebral blood flow did not differ between groups	MwA subjects have cortical hyperresponsiveness to visual stimulus, suggesting a direct connection between cortical hyperresponsiveness and aura itself
Zhang et al., 2017 [40]	116 migraine pts (56 MwA, 60 MwoA) during interictal period, 54 HC	3 T MRI (including 3D pseudo-continuous ASL)	- MwA pts with high white matter hyperintensities load had lower CBF - No association between white matter hyperintensities load and CBF was seen in HC and MwoA pts	White matter hyperintensities in MwA may be related to alterations in resting CBF (or viceversa)
Hu et al., 2019 [41]	24 CM pts; 24 HC	3 T MRI (including 3D pseudo-continuous ASL-fMRI and BOLD-fMRI images)	NVC biomarkers were: - lower in L inferior parietal gyrus, L superior marginal gyrus, and L angular gyrus - higher in R superior occipital gyrus, R superior parietal gyrus, and precuneus These areas were related to visual or sensory information processing ALFF-CBF: - in R superior parietal gyrus positively correlated with disease history while R precuneus negatively correlated with migraine persisting time fALFF-CBF: - in L superior marginal gyrus and in L angular gyrus were negatively related to headache frequency and positively related to health condition	Multi-modal MRI could be used to detect NVC dysfunction in CM pts to assess the impact of chronic pain to the brain
Park et al., 2022 [42]	58 migraine pts (11 MwA, 47 MwoA) during interictal period	3 T MRI (including 3D pseudo-continuous ASL)	Pts with MwA vs MwoA present: - no differences in the network measures of global structural connectivity - differences in global functional connectivity (lower assortative coefficient)	Low network global assortativity may result in more vulnerable, easily disrupted, highly synchronized networks and, therefore more excitable brain connectome in MwA
Stankewitz et al., 2021 [43]	- 12 MwoA pts during attack (within 6 h after the beginning) rescanned every 1–4 days (final recording within 48 h before the subsequent attack)	3 T MRI (including pseudo-continuous ASL)	- Cyclic changes of brain perfusion in the limbic circuit, with the highest perfusion during attack - Increase of hypothalamic connectivity to the limbic system over the interictal interval towards the attack, then collapsing during the headache phase	The hypothalamus seems to act as a metronome of internal processes, able to control limbic pathways, and migraine attacks may represent the result of the lost hypothalamic inhibition on the limbic circuit

*MwA*  Migraine with aura, *pts*  Patients, *h* hours, *MRI* Magnetic resonance imaging, *TOF-MRA*  Time-of-flight magnetic resonance angiography, *ASL*  Arterial spin-labeling, *R* Right, *DSC* Dynamic susceptibility contrast, *L* Left, *PWI* Perfusion Weighted Imaging, *CBF*  Cerebral blood flow, *DWI*  Diffusion weighted imaging, *pt*  patient, *CM*  Chronic migraine, *FLAIR*  Fluid-attenuated inversion recovery, *MwoA*  Migraine without aura, *min*  minutes, *mg* milligrams, *CGRP*  Calcitonin Gene Related Peptide, *HC* Healthy controls, *CDS*  Cortical spreading depression, *VSS*  Visual snow syndrome, *BA*  Brodmann area, *HAMD*  Hamilton Depression Scale, *VLSQ8*  Visual Light Sensitivity Questionnaire-8, *RS*  Resting state, *NDPH*  New daily persistent headache, *BOLD*  Blood oxygenation level dependent, *fMRI*  functional MRI, *NVC*  Neurovascular coupling, *ALFF*  Amplitude of low-frequency fluctuation, *fALFF*  functional amplitude of low-frequency fluctuation

#### ASL investigation during aura and headache phases of migraine with aura attacks

About 30% of patients with migraine reports fully reversible focal neurological symptoms, constituting the so-called aura, gradually spreading in about 5 min, most often before the headache onset (more rarely starting along with headaches), until slowly disappearing between 5 to 60 min.

Visual symptoms, either negative or positive, are the most frequently reported in the course of aura, followed by somatosensory symptoms (frequently manifesting as cheiro-oral paraesthesias) and dysphasic symptoms respectively in about one third and 10% of patients [44, 45]. Nevertheless, patients may rarely report other higher cortical dysfunctions such as memory disturbances, prosopagnosia, dyscalculia and manual dyspraxia in the course of aura phenomenon. The cortical spreading depolarization (CSD), a wave of initial increase, followed by a sustained decrease, was invoked as the pathophysiologic mechanism underlying the aura phenomenon based on well-known electrophysiological studies on rabbit brain [46]. Only several decades later, the pioneering studies of Olesen and colleagues demonstrated, using 133Xe-SPECT during aura phase of provoked or spontaneous migraine attacks, a cortical oligemia spreading from occipital areas to the anterior regions over 15–45 min, not congruent with large cerebral arteries vascular territories [47, 48]. Interestingly, the regional hypoperfusion involving cerebral hemisphere during the aura symptoms kept being detectable by SPECT sequences also after 30 and 150 min from the onset of spontaneous migraine with aura and the same cerebral regions were then overwhelmed by hyperemia [49]. Subsequently, Hadjikhani and colleagues by means of BOLD-fMRI experiments in patients with migraine during the aura symptoms showed that perfusion changes, far from being the *“primum movens”* of aura, resulted from autoregulatory mechanisms secondary to the neuronal spreading depression [50–52]. These findings stimulated the interest of researchers and clinicians so that several ASL studies have been conducted to explore the perfusion pattern of aura phenomenon in order to elucidate the underpinning mechanisms and to improve the differential diagnosis between the manifestations of aura spectrum and cerebral ischemic events.

Among these studies, patients with migraine were investigated with 2D-pASL within 6 h from the onset of aura phenomenon to identify CBF changes correlated with the diagnosis of migraine with aura (MwA). The results showed asymmetrical hypoperfusion, predominantly in the parieto-occipital regions, not confined to specific vascular territories [52]. The aura-related perfusion changes were associated, in the majority of patients (74%), with intracranial vasculature vasospasm as detected by TOF-MRA images. Nevertheless, both perfusion and vasculature features were only qualitatively evaluated based on a mere visual assessment [16]. Pathophysiological and clinical insights on aura-related cerebral hypoperfusion could be even more relevant in complex aura phenotypes (such as hemiplegic migraine, atypical aura or confusional migraine attacks) often representing a diagnostic challenge, difficult to be differentiated from acute ischaemic strokes. Focusing on hemiplegic migraine attacks, a pattern of cerebral hypoperfusion involving the hemisphere contralateral to motor dysfunction was detected since the first ASL approaches [17]. However, over time, specific and accurate data arose from studies employing ASL technique evolutions. For instance, using 3D-pcASL, abnormal CBF has been demonstrated in paediatric patients with MwA manifesting complex aura phenotypes [18]. Interestingly, patients investigated within 14 h from the onset of the aura exhibited a decreased CBF, involving the hemisphere contralateral to aura symptoms, whereas after 17 h from the onset of the aura, an increased CBF was highlighted in the same areas, independently from the symptoms or the persistence of either aura or headache at the time of the scan [18]. This biphasic perfusion evolution characterized by an initial regional hypoperfusion followed by hyperperfusion, seems to represent the typical pattern of MwA attack since it has been observed also in patients experiencing typical visual MwA when investigated after the onset of aura phenomenon as well as when explored several hours after the aura resolution during the headache phase [19–21]. The shift from regional cerebral hypoperfusion to hyperperfusion (due to delayed arrival of labelled blood in the territories involved) justify the so-called “arterial transit artefact” in patients explored during the transition from the aura to the headache phase, as already demonstrated in the early reperfusion following intravenous thrombolysis in patients with ischaemic stroke [21].

While the presence of a biphasic CBF changes involving aura and early headache phases is an indisputable fact, the time-course of the perfusion phenomenon seems difficult to be consistently delineated. Therefore, 3D-pcASL quantitative and qualitative analyses, along with MRA and SWI qualitative analyses, have been conducted in patients with MwA or hemiplegic migraine within 12 h from symptoms onset. Consistently with previous studies, initial hypoperfusion (with concomitant apparent constriction of distal vessels on MRA and deoxygenated blood in cerebral veins on SWI) followed by a rebound hyperperfusion in the affected hemisphere were demonstrated [23]. Focusing on the time-course of perfusion changes, the duration of cerebral regional hypoperfusion lasted over 11 h (since the aura onset) in the majority of patients, although more rapid transition to the rebound hyperperfusion could be noticed in some patients. Interestingly, a discordance between increased regional CBF and reduction of vessel calibres was observed during the hyperperfusion in a small percentage of patients, suggesting reversible disturbances of neurovascular coupling (the mutual relation between neuronal activity and perfusion) during the transition from aura and headache phases.

Considering the cerebral topography of the biphasic hypoperfusion-hyperperfusion phenomenon, the involvement of posterior regions during the attack of sporadic hemiplegic migraine was highlighted in an early anecdotal report [17]. Subsequently, a pASL study with a larger sample-size confirmed that occipital and parietal cortices were the most frequently encompassed by hypoperfusion in patients with MwA compared to patients with MwoA, whit a significant correlation between the hyperperfusion magnitude and the complexity of aura phenotype [24]. Although limited, these findings suggest that i) the shift from regional cerebral hypoperfusion to hyperperfusion may occur in some patients sooner than expected according to the findings of previous ASL studies ii) the time-gap between the clinical onset of aura and MRI images acquisition is crucial more than the actual presence of symptoms at the time of imaging scan, considering the risk of detecting aura-related hypoperfusion also in the course of the following headache, when the aura symptoms are clinically extinguished iii) posterior cerebral areas, especially parietal and occipital cortices, are preferentially involved by perfusion changes in the course of both aura and headache phases of migraine attacks.

Altogether the above-mentioned ASL findings, beyond the insight provided about the pathophysiological mechanisms underlying the different phases of migraine cycle, give some critical pieces of advice with potential impact in the clinical practice. First, the presence of hypoperfusion during the first phases of MwA attacks, especially in patients with hemiplegic migraine, may suggest avoiding triptans during the aura symptoms in these patients due to the triptans vasoconstrictive properties and related concerns about ischaemic events. Moreover, ASL techniques, detecting in patients presenting with aura symptoms the presence of biphasic CBF changes involving vascular territories belonging to different vessels in the absence of DWI abnormalities, may allow us to confute alternative diagnoses such as ischaemic attacks and epileptic seizures.

#### ASL investigation during migraine without aura attacks

It should be underlined that there is a scarcity of brain perfusion studies based on ASL sequences focusing on the headache phase of MwoA attacks and those conducted have shown inconsistent results. Indeed, it has been reported, by anecdotic observations, a significant relative hypoperfusion in the bilateral median thalamic structures (including hypothalamus) as well as in the frontal cortex during the headache phase of MwoA when compared to the interictal phase [25]. Interestingly, after 30 min from rescue therapy with rizatriptan, the observed hypoperfusion partially normalized. These findings support the involvement of hypothalamus and surrounding brain structures as a critical mechanism in the pathogenesis of migraine attacks, as widely demonstrated by means of BOLD investigations [53].

Nevertheless, other observations tend to be in line with the pioneering observations of Weiller showing, by means of H2^15^O PET, a higher regional CBF values in the dorsal pons during a spontaneous MwoA attack, persistent after sumatriptan-related pain relief, compared to the pain-free state [54]. In particular, a multiparametric study evidenced a significant CBF increase in the dorsolateral pons (ipsilaterally to the headache side) concomitantly with increase in creatine levels (in the absence of changes in glutamate concentration), during provoked MwoA attacks (by CGRP or sildenafil) compared to the interictal period and to healthy subjects [26]. Contrariwise, by using 3D-pcASL, no global or regional perfusion differences were identified during spontaneous migraine attacks and migraine-free phases [27].

It should be underlined that the results of the above-mentioned longitudinal studies, aimed to investigate different phases of migraine attacks in the same patient (e.g. ictal versus interictal scans), may be potentially biased by the well-known influence of caffeine intake, tobacco smoking, and hematocrit changes on CBF, as well as by the physiologic circadian variation of CBF values.

Despite the paucity of studies, the above-mentioned data highlight the momentous involvement of dorsolateral pons and hypothalamus in the headache phases of migraine attacks. However, they are not able to disentangle which structure can assume the role of “migraine generator” or, whether, alternatively, the activation of both hypothalamus and dorsolateral pons could represent an epiphenomenon of the persistence of hypothalamus involvement (started in the so-called preictal phases of migraine attacks) and the migraine-related pain perception, respectively [55–57].

#### ASL investigation during the interictal period

Similarly to the lack of converging evidences about the presence of perfusion abnormalities during the headache (i.e. ictal) phases in patients experiencing MwoA, many discrepancies emerged from ASL studies investigating the interictal period of both patients with Mwa and patients with MwoA.

Patients with episodic migraine (both with and without aura) showed extrastriate cortex (V5 area) and superior temporal gyrus hyperperfusion compared to healthy subjects when explored by means of 2D-pcASL during the interictal period [28]. Interestingly, the perfusion abnormalities were more pronounced in MwA patients [28]. The involvement of posterior brain areas, in the terms of asymmetries of blood flow in the posterior cerebral artery territories, has been also demonstrated, by exploring a large sample of 170 subjects, in MwA patients (but not MwoA) during the interictal period [29]. Interestingly, the hypoperfusion in the territories supplied by the posterior cerebral artery was significantly associated with a higher prevalence of abnormalities of the circle of Willis.

A significant increase in regional CBF has been demonstrated in the primary somatosensory cortex (S1) in patients with migraine experiencing ictal cutaneous allodynia investigated during the interictal period (subjects were headache-free from at least 48 h at the time of scan acquisition) [31]. Moreover, the authors found a positive correlation between regional CBF degree and headache attack frequency, as already observed also in a cohort of paediatric and adolescent patients with migraine [32]. Increased regional CBF has been also demonstrated by means 3D-pc-ASL in the temporal areas (Brodmann 38), supposed to represent a convergence zone of information from auditory, somatosensory, visual, and olfactory inputs and, therefore, involved in multisensory integration [33].

Finally, interictal regional CBF abnormalities have been recently investigated by means of 3D-pcASL in a large cohort of patients with migraine. After whole brain voxel-based CBF comparison between patients with migraine and healthy subjects, the areas showing significant differences between the groups were extracted as ROIs to compare the normalized regional CBF values among all the subjects [34]. Over and above the visual, somatosensory and multisensory integration cortices observed in the previous studies, higher CBF levels in superior frontal and postcentral gyri and cerebellum, and lower CBF levels in the bilateral middle frontal gyrus, thalamus and medioventral occipital cortex have been demonstrated in patients with MwA compared with patients with MwoA and healthy subjects. Interestingly, based solely on CBF values of these six cerebral regions, by using a machine learning approach, it was possible to correctly classify more than 80% of patients with MwA or MwoA [34].

In this scenario, although aimed to investigate the CBF pattern underpinning visual snow, a syndrome characterized by the presence of continuous and unremitting visual disturbances often, but not exclusively, found as migraine comorbidity [30, 58], a very recent 3D-pcASL study is worth to be mentioned due to the enrolment of patients with MwA and MwoA. While a higher regional CBF encompassing an extensive brain network has been demonstrated in patients with visual snow (regardless of concomitant migraine) during both’snow-like’ visual stimuli and rest condition, a post-hoc analysis has suggested that the involvement of bilateral cuneus, posterior cingulate cortex, primary auditory cortex, fusiform gyrus, and cerebellum might be more closely related to the interictal phases of migraine. Visual symptoms, and specifically visual sensitivity to light (explored by Visual Light Sensitivity Questionnaire-8), along with migraine frequency have demonstrated a positive correlation with reduced regional CBF of frontal areas in a wide sample of patients with MwoA compared with healthy subjects [35]. Moreover, frontal ROI were characterized by a disrupted CBF connectivity with several cortical (i.e., contralateral frontal and angular gyri and calcarine cortex) and subcortical (i.e., putamen and caudate) brain areas, further supporting the involvement of an “advanced neurolimbic visual pain network” in patients with MwoA as well as in patients with MwA [59].

A reduced CBF of nucleus accumbens (identified as ROI using a specific nucleus accumbens perfusion mask) has been demonstrated by 3D-pcASL in patients with chronic migraine during the interictal period when compared with healthy subjects [36], further corroborated by the ROC curve analysis enabling the identification of patients with chronic migraine from healthy subjects (sensitivity of 50.00% but with a specificity over 90%). Apart from the known role played by the nucleus accumbens in pain modulation and reward/addiction mechanisms, no specific information have been reported about the presence of medication overuse headache in these patients. Moreover, the lack of a comparison group (e.g., patients with episodic migraine) made not possible to clarify the specificity of nucleus accumbens hypoperfusion in chronic migraine. CBF changes have been recently investigated by the same research group in patients with chronic migraine compared with healthy subjects, using the novel multi-delay-3D-pcASL approach, showing increased regional CBF and arterial cerebral blood volumes in bilateral ventral lateral nuclei of thalamus [37]. Being the thalamus a relay centre of ascending trigemino-cortical nociceptive information, characterized by increased activation during migraine attacks as well as by abnormal functional connectivity in the interictal period in patients with episodic migraine [60], the authors suggested that the chronification process, towards a never-ending attack, could be underpinned by a constant increase of neuronal thalamic activation and, in turns, of regional CBF. However, this riveting hypothesis is burdened by a low sample size and spatial resolution making impossible to clearly discriminate among thalamic nuclei (being the ASL imaging resolution of about 1.6 mm × 1.6 mm × 4.0 mm).

Totally different are the results from three recent studies, finding no CBF changes in both patients with MwoA and MwA [38–40], although a significantly lower global CBF characterizes patients with MwA with high white matter hyperintensities load explored during interictal period when compared to healthy subjects [40]. Albeit the findings do not allow a univocal interpretation, the authors argued that the well-known CBF fluctuations observed during the MwA attacks might affect microvascular haemodynamic leading to microischaemic injury that can be observed also during the interictal period. Nevertheless, the possibility that the decreased CBF could be the result, rather than the cause, of white matter abnormalities in patients with MwA cannot be excluded, to date [40].

The evidences of both impaired cerebral perfusion and functional neuronal changes tend to support the hypothesis of an altered relationship between neuronal activity and cerebral perfusion maps (i.e. neurovascular coupling) during the different phases of migraine cycle. On the other hand, neurovascular coupling abnormalities have been demonstrated in patients with chronic migraine investigated by using 3D-pcASL technique [41]. Specifically, significantly lower neurovascular coupling indices in inferior parietal, superior marginal and left angular gyri, as well as significantly higher neurovascular coupling indices in precuneus, superior occipital and parietal gyri were identified, probably underpinning migraine-related abnormalities in visual and sensory processing.

The 3D-pcASL has been recently employed, along with DWI sequences, to investigate global functional and structural brain connectome parameters in patients with MwoA patient compared to patients with MwA [42] showing no differences in network measures of global structural connectivity but significantly lower assortative coefficient in patients with MwA compared to patients with MwoA. Based on the fact that assortativity coefficient is a parameter of network resilience able to quantify the mutually interconnection among cortical hubs (so-called nodes), networks with low assortativity, as observed in patients with MwA, are more vulnerable to insult, easily disrupted, and more synchronized than those with high assortativity [61]. Unfortunately, the low sample size as well as the absence of a healthy subjects group strongly affect both the specificity and the generalizability of the results.

Although the above-mentioned studies suffer from several limitations such as differences in patients homogeneity, disparities in ASL techniques (with consequent divergences in labeling schemes, background suppressions, dimensional resolutions, etc.), as well as in methodological procedures (e.g. whole-brain analysis or ROI-based approaches), altogether they support disease-specific functional changes in trigemino-cortical pathway and in brain regions known to be involved in somatosensory, auditory and visual processing suggesting abnormal pain perception and modulation as well as dysfunctional mechanisms of multisensory integration in both patients with MwoA and MwA, also during the interictal phase. To the same extent, ASL findings, consistently with data from advanced neuroimaging literature [62], further corroborate the critical role of brain structures related to cortical spreading depression ignition and propagation also between MwA attacks.

#### ASL investigation during the preictal phase

In the last years, besides with the insight about the differences within the phases of migraine cycle the preictal phase of migraine attacks has been specifically investigated with even more advanced ASL approaches as well as using methodologically rigorous study paradigms. Unfortunately, due to the unpredictability of migraine attacks, it is quite difficult to collect MRI scans during the period immediately prior to migraine attacks, frequently only by chance and, therefore, in a low sample of patients.

By means of 3D-pcASL, immediately before the onset of headache in patients with MwoA (within at least 24 h), a decreased regional CBF has been observed in the lateral hypothalamus that was also characterized by a reduced functional connectivity with several regions within the pain processing pathway (e.g. midbrain periaqueductal grey, dorsal pons, rostral ventromedial medulla and cingulate cortex) [38]. These findings are in line with literature data supporting the role played by the hypothalamus in migraine attack ignition previously suggested by both PET and fMRI studies showing an increased hypothalamic activity during the hours preceding the headache of the migraine attack [56, 57].

The rhythmicity of the cerebral perfusion and changes of hypothalamic functional connectivity over migraine cycle have been explored by daily longitudinal intra-individual scanning (82 scans to cover the course of migraine cycles of twelve patients with migraine) [43]. Interestingly, during the headache phases, the highest perfusion of the limbic circuit (i.e., insula and nucleus accumbens) was detected along with a collapse of functional connectivity between the hypothalamus and the limbic areas. More in depth, the functional connectivity between hypothalamus and limbic structures progressively increased, over the migraine cycle towards the next attack, up to rapidly drop during the headache phase. According with these data, while the hypothalamus seems to act as a metronome of internal processes, able to control limbic pathways, the migraine attacks may represent the result of the lost hypothalamic inhibition on the circuit.

Whether the functional and perfusion hypothalamic abnormalities may represent the correlates of the brain changes closely preceding the headache phases of migraine attacks, reflecting modulatory input to the trigeminovascular system from several cortical and subcortical areas, or if they represent the neuronal and perfusion correlates of preictal symptoms is still matter of debate, although the current trend to accept that premonitory symptoms as due to hypothalamic dysfunction seems to be premature [63]. Future insight on postdrome phase could further improve our knowledge about migraine cyclicity shedding a light on the mechanisms underlying not only the ignition of migraine attacks but also the self-limitation of migraine attacks in a way characterizing the typical migraine attack duration (i.e., the well know duration of about 4–72 h) and that, on the other hand, may set up the neuronal patterns towards the interictal changes predisposing the next migraine attack.

### Final considerations

Taken together, the data emerging from the ASL studies in patients with migraine allow to achieve some conclusions:

- 1) During MwA attacks the areas congruous with aura symptoms are characterized by an altered cerebral perfusion, exceeding the boundaries of a single vascular territory, with a biphasic trend dominated by an initial hypoperfusion (persisting even during the first hours of the headache attack) followed by an hyperperfusion phase. However, future studies are mandatory to determine the duration of the hypoperfusion, over different phases of migraine cycle, as precise as possible. Nevertheless, beyond the role of ASL techniques to explore migraine pathophysiological mechanisms, they could represent a valuable clinical tool to be applied in the differential diagnosis of migraine aura from acute ischemic strokes (in particular in the cases of atypical aura, hemiplegic migraine, confusional migraine, HANDL).
- 2) Although perfusion abnormalities have been observed in the visual associative and primary somatosensory cortices as well as in areas involved in the processing and modulation of the painful experience, the scarcity of data does not allow univocal and conclusive insight on regional CBF changes found in the intercritical period, as witnessed by the conflicting results and most likely due to several methodological issues. Among these, i) different ASL techniques adopted in the perfusion studies, from the most pioneering and rudimental to the most advanced and accurate approaches, up to the more complete multi delay-3D-pcASL which has been only recently sufficiently developed for clinical studies; ii)) heterogeneous patients samples (e.g. groups of patients including different migraine phenotype, with or without concomitant preventive treatments, with no reference to the intake of medications for the acute phase).
